# Long-term health-related quality of life in patients on home mechanical ventilation

**DOI:** 10.1186/s12890-022-02236-z

**Published:** 2022-11-22

**Authors:** Carla Ribeiro, Cristina Jácome, Luísa Castro, Sara Conde, Wolfram Windisch, Rui Nunes

**Affiliations:** 1 Pulmonology Department – Centro Hospitalar de Vila Nova de Gaia/Espinho, Rua Conceição Fernandes S/N 4434-502, Vila Nova de Gaia, Portugal; 2 Center for Health Technology and Services Research (CINTESIS), Faculty of Medicine, University of Porto, Alameda Prof. Hernâni Monteiro, 4200-319 Porto, Portugal; 3 MEDCIDS - Department of Community Medicine, Information and Health Decision Sciences, University of Porto, Alameda Prof. Hernâni Monteiro, 4200-319 Porto, Portugal; 4 School of Health of the Polytechnic of Porto, R. Dr. António Bernardino de Almeida 400, 4200-072 Porto, Portugal; 5 Department of Pneumology, Faculty of Health/School of Medicine, Cologne Merheim Hospital, Kliniken Der Stadt Köln gGmbH, Witten/Herdecke University, Ostmerheimer Str. 200, 51109 Köln, Germany; 6 Faculty of Medicine, University of Porto, Alameda Prof. Hernâni Monteiro, 4200-319 Porto, Portugal

**Keywords:** Noninvasive ventilation, Patient reported outcome measures, Quality of life, Survival analysis

## Abstract

**Background:**

It is fundamental to optimize and retain health-related quality of life (HRQoL) in the long term in patients with home mechanical ventilation (HMV). Therefore, this study aimed to evaluate the evolution of the HRQoL in patients already established on HMV across a period of 5 years and whether the HRQoL is associated with mortality.

**Methods:**

This was a 5-year longitudinal cohort study conducted in an Outpatient Ventilation Clinic. Consecutive patients on HMV for at least 30 days responded to the Severe Respiratory Insufficiency (SRI) questionnaire at inclusion and again at 5 years.

**Results:**

A total of 104 patients were included (male 56.7%, median age 69 [P25;P75] [61;77] years). Almost half of the patients had COPD (49.0%). Patients were on HMV for a median of 43.5 [22;85.5] months, with overall good adherence (median 8 [6;9] daily hours). Fifty-seven (54.8%) patients were alive at 5 years. In surviving patients, the only difference with statistical significance was in the attendant symptoms and sleep subscale, with patients scoring 7.1 [-4.5;25] points higher in the final questionnaire (*p* = 0.002). Survivors had significantly better scores in the SRI at inclusion than deceased patients (median 59.6 [49.2;71.7] vs 48.7 [38.4;63.2]; *p *= 0.004).

**Conclusions:**

These results shows that HRQoL remains stable in surviving patients with HMV at five years. It also suggests that SRI can be of important prognostic value and help predict the terminal phase of the disease course in patients with long-term HMV.

**Supplementary Information:**

The online version contains supplementary material available at 10.1186/s12890-022-02236-z.

## Background

Home mechanical ventilation (HMV) is indicated in patients with chronic respiratory failure (CRF) of different causes and its utilization in the last decades has been increasing both due to widening indications and improved health care setting organization [1]. While in the Eurovent trial the estimated prevalence of HMV in Europe was 6.6/100000 people in 2001–2002, [2] a more recent study described a prevalence up to 38/100000 inhabitants in two Swiss regions and a 2.5-fold increase in HMV prescription since 2000 [3].

One of the main goals of HMV is to improve survival and halt disease progression, thus during the last decades, trial endpoints have been primarily linked with outcomes such as survival improval in patients with neuromuscular diseases, [4] restrictive chest wall disorders, [5] and most recently, in stable chronic obstructive pulmonary disease (COPD) [6]. Recently, patient-reported outcomes measures (PROMs) have been listed more commonly endpoints in clinical research. This is mostly related with the need of promoting more people-centered care, which has become a growing priority in the European governments to improve the quality of care and the responsiveness to patients’ expectations [7].

Health-related quality of life (HRQoL) questionnaires are multidimensional tools that explore aspects of patients’ lives that assess physical, mental and social well-being, which are not usually covered by other diagnostic tools. They are an invaluable asset to understand how disease affects a patient’s life, and they should be sensible to changes related to progression of disease or treatment interventions and have predictive purposes [8]. Their importance is so clear that health commissioners are now routinely seeking this information to make relevant health economic policies [9]. The most widely used specific tools for assessing HRQoL in long-term HMV users (independent of the cause of CRF) are the Severe Respiratory Insufficiency (SRI) Questionnaire, [10] and the Maugeri Respiratory Failure (MRF-26) Questionnaire [11]. A recent comparison showed that both SRI and MRF-26 were reliable in patients receiving HMV, but that the SRI was superior in assessing psychological health impairments [12]. In addition, a cohort study found that SRI score was associated with mortality of patients with long-term HMV and proposed the use of SRI in the daily clinic as part of patients’ follow-up [13].

HRQoL assessments are now included in most of the trials measuring the health impacts of HMV, although over short to medium timeframes (12 months or less) [14–17]. It is uncertain whether there is a point in the disease trajectory where burdens of HMV outweigh benefits, overcoming the initial quality of life improvement. Repeated measures over time are vital to ascertain the evolution of HRQoL in ventilated patients [9]. A prospective 6-year study found that the total SRI score and the scores in four of the seven subdomains improved in the majority of the patients treated with long-term HMV [18].

The aim of this study was to evaluate the differences of the HRQoL measured by the SRI in patients already established on HMV at two points in a 5-year period in order to ascertain whether HRQoL is maintained in the long term. A secondary objective was to evaluate whether the HRQoL has a prognostic value for mortality in these patients.

## Methods

### Study design

This was a 5-year longitudinal cohort study conducted between January 2016 and January 2022. Patients were recruited in an Outpatient Ventilation Clinic of the Pulmonology Department at Centro Hospitalar de Vila Nova de Gaia/Espinho (Portugal), a tertiary care teaching hospital. Ethical approval was obtained from the Hospital Ethics Committee (Ref 92/2015 and 163/2020) and written consent was obtained from all included patients. This study was reported according to STROBE (STrengthening the Reporting of OBservational studies in Epidemiology) guidelines [19].

### Participants

Adult patients with CRF, from a wide variety of causes, already established on HMV for at least 30 days were included. Exclusion criteria were refusal to participate, unable to understand or answer the questionnaire or an exacerbation in the previous 30 days. All consecutive patients who had a scheduled medical appointment between January and June 2016 and fulfilled the eligibility criteria were invited to participate.

### Data collection

At recruitment, demographic information (age, sex) and SRI questionnaire were obtained from the patient. Daytime arterial blood gas (ABG) analysis was obtained according to standard recommendations in sitting patients without ventilation, with the current oxygen flow provided [20]. Clinical data [body mass index (BMI), disease, time with HMV, ventilation interface, humidifier] were collected from the electronic hospital records. Pulmonary function test data was obtained from the electronic clinical records if obtained at least 12 months previous to the beginning of the study. Ventilation parameters and daily usage were recorded by the readout of the ventilator’s built-in software (*ResMed AirView*® and *Philips EncoreAnywhere*® platforms), obtained during a scheduled medical visit. At 5 years, patients completed the SRI again during scheduled medical visits. Time to complete the questionnaire was recorded at both times.

If patient died in the study period, date of death was obtained from the electronic hospital records or national patient information registry (if death was outside the hospital).

### Questionnaires

The SRI Questionnaire [10] is a self-administered questionnaire containing 49 items that patients answer on a 5-point Likert-scale (1: completely false; 2: quite false; 3: partly true/partly false; 4: quite true; 5: completely true) according to how true each statement has been for them in the preceding week. When patients were unable to answer by themselves, the questionnaires were completed with the help of the caregiver or a family member.

The questionnaire contains 7 HRQoL subscales: respiratory complaints (SRI-RC)—8 items, physical functioning (SRI-PF)—6 items, attendant symptoms and sleep (SRI-AS)—7 items, social relationships (SRI-SR)—6 items, anxiety (SRI-AX)—5 items, psychological well-being (SRI-WB)—9 items and social functioning (SRI-SF)—8 items. The final score for each subscale is calculated, after recoding certain items, by the corresponding percentage. The summary score (SRI-SS) is obtained by calculating the arithmetic mean of the subscale scores, in such a way that this calculation is not possible if any of the subscale scores were missing. The summary score and subscales range from 0 to 100 with a high score indicates a good HRQoL, while a low score indicates a poor HRQoL [10]. The Portuguese SRI version was used, which has been validated in 93 patients with CRF of different causes (most commonly COPD, obesity hypoventilation syndrome and restrictive chest wall disorders) and presented good psychometric properties with values for Cronbach’s alpha above 0.70 for most subscales and 0.84 for the summary scale [21].

### Data analysis

Patients were categorized into five categories depending on the diagnosis: COPD (irrespective of overlap with obstructive sleep apnea), restrictive chest wall disorders (RCWD), obesity hypoventilation syndrome (OHS), neuromuscular disorders (NMD) and other pathologies. In order to compare baseline patients’ demographic, clinical and ventilation characteristics, and SRI questionnaire patients were divided by COPD and other diseases.

Categorical variables were described by absolute and relative frequencies (n, %). Normality of quantitative variables was verified by visual inspection of histograms. Since most variables deviated from normality, quantitative variables were described using median and 25 and 75 quartiles [P25-P75]. The Wilcoxon test was employed to compare SRI summary scale and subscale scores at inclusion and after 5 years. The Mann–Whitney test was used to compare scores between groups of patients (deceased/survivors). Proportions of patients survived/deceased were compared between diseases using the Chi-square test. In contingency tables larger than 2 × 2 where more than 20% of cells had expected counts below 5, the Fisher-Freeman-Halton Exact test was used instead. Spearman correlations (rho) were computed to assess the association between SRI subscales.

The Kaplan–Meier survival analysis was applied to the data and survival curves are presented for each pathology. The log rank test was used to compare factor levels (the pathology categories), which tests the equality of the survival functions considering all the points in time weighted equally. The date of the application of the SRI for the first time was set as the initial time for the analysis. Patients were followed up to death or censured at 60 months.

In order to explore the ability of the baseline SRI in discriminating survivors from deceased, a receiver operating characteristic (ROC) curve was computed and an estimate (with 95% CI) of the area under the ROC curve (AUROC) was obtained. This curve relates sensitivity—ability to predict the patient will pass away —and specificity—ability to predict the patients that will survive—for each cutoff of the discriminating index, which is the SRI baseline. The Youden Index was applied to elect the best cutoff on the SRI baseline for discriminating deceased from survivors, which corresponds to calculating the maximum of (Specificity + Sensitivity) for all the points in the ROC curve [22].

Statistical computations were performed with IBM SPSS Statistics for Windows, Version 27.0 (Armonk, NY: IBM Corp.). In all tests, statistical significance was assumed when *p* < 0.05.

## Results

### Participants

From a total of 171 patients followed in the clinic and 156 patients on HMV, 104 patients fulfilled the inclusion criteria and were included, whose demographic, clinical and ventilation characteristics are described in Table 1. The majority were male (56.7%), with a median age of 69 years and almost half of the patients had COPD (49.0%). Patients were on HMV for a median of 43.5 [22;85.5, min 3 max 190] months and had overall good adherence (median 8 [6;9] daily hours), with the vast majority using the ventilator during night-time (94.2%). The most commonly used interface was oronasal mask (73.1%), followed by nasal mask (26.9%) with no patients on invasive ventilation.

**Table 1:** Baseline patients’ demographic, clinical and ventilation characteristics, and Severe Respiratory Insufficiency (SRI) questionnaire results (*n* = 104)

**Male, n (%)**	59 (56.7)
**Age, years**	69 [61; 77]
**BMI**^**a**^**, Kg/m**^**2**^	30.5 [25.3;37.3]
**Disease, n (%)**	
** COPD**	51 (49.0)
** RCWD**	22 (21.2)
** OHS**	21 (20.2)
** NMD**	7 (6.7)
** Other**	3 (2.9)
**FEV1 (% predicted)**^**b**^	42.5 [27.8;57.7]
**FVC% (% predicted) **^**b**^	59.1 [46.9;72.5]
**pH**	7.4 [7.4;7.4]
**pO2 mmHg**	69.4 [64.5;77.4]
**pCO2 mmHg**	46 [42.9;49.1]
**HCO3 mmol/L**	28.3 [26.5;30.3]
**Months on HMV**	43.5 [22;85.5]
**HMV Usage (hours/day)**	8 [6; 9]
**Baseline SRI questionnaire**	
SRI-RC: respiratory complaints	59.4 [43.8;74.2]
SRI-PF: physical functioning	45.8 [26;66.7]
SRI-AS: attendant symptoms and sleep	50 [39.3;64.3]
SRI-SR: social relationships	75 [62.5;91.7]
SRI-AX: anxiety	40 [25;65]
SRI-WB: psychological well-being	55.6 [41.7;71]
SRI-SF: social functioning	56.3 [40.6;75]
SRI-SS: summary scale	53.3 [44.9;68.2]

Data are presented as median [percentile 25, percentile 75] unless otherwise indicated

*Abbreviations*: *COPD* chronic obstructive pulmonary disease, *OHS* obesity-hypoventilation syndrome, *RCWD* restrictive chest wall disorders, *NMD* neuromuscular disorders, *BMI* body mass index, *HMV* home mechanical ventilation, *FVC* forced vital capacity, *FEV1* forced expiratory volume in one second, *IPAP* inspiratory positive airway pressure, *EPAP* expiratory positive airway pressure

^a^2 missing

^b^6 missing

In the baseline SRI questionnaire, patients scored a little above the middle of the scale range (53.3) with highest median values for the social relationship’s subscale (75) and lowest for the anxiety subscale (40). Except for a lower SRI anxiety subscale (35 [20;65] vs 45 [35;70], *p* = 0.016), there were no significant differences in SRI subscales and summary scale between COPD and other diseases. The majority of the questionnaires were self-administered. Thirty patients (28.8%) required help because they were unable to read, did not bring their reading glasses or were physically too disabled to write. On average, patients took approximately 8 min to complete the questionnaire.

### HRQoL of survivors at baseline and at 5 years

From the 104 patients, 57 (54.8%) patients were alive at 5 years. The baseline patients’ demographic, clinical and ventilation characteristics of survivors and deceased patients is described in supplementary table S1. Of the 57 survivors, 54 answered the SRI questionnaire at 5 years (2 patients were too disabled to answer and 1 patient declined to answer). The results of the questionnaire at the two time-points (at inclusion and 5 years) and respective differences are described in Table 2.

**Table 2:** Severe Respiratory Insufficiency (SRI) results of the survivors at inclusion and at 5 years (*n* = 54)

**Questionnaire subscale**	**SRI at inclusion** **(*n* = 54)**	**SRI at 5 years** **(*n* = 54)**	**Median difference**	***p*-value**
**SRI-RC: respiratory complaints**	65.6 [46.9;81.3]	73.4 [52.3;84.4]	+ 3.1 [-8.7;15.6]	0.104
**SRI-PF: physical functioning**	56.3 [37.5;70.8]	50 [29.2;76]	-8.3 [-17.7;12.5]	0.455
**SRI-AS: attendant symptoms and sleep**	50 [39.3;64.3]	58.9 [42.9;82.1]	+ 7.1 [-4.5;25]	0.002*
**SRI-SR: social relationships**	75 [62.5;91.7]	83.3 [69.8;92.7]	+ 4.2 [-12.5;20.8]	0.199
**SRI-AX: anxiety**	45 [33.8;70]	60 [25;76.3]	+ 2.5 [-11.3;21.3]	0.379
**SRI-WB: psychological well-being**	58.3 [47.1;72.2]	65.3 [44.4;81.3]	+ 5.6 [-9;20.1]	0.144
**SRI-SF: social functioning**	65.6 [46.1;84.4]	65.6 [45.9;84.4]	-2.2 [-18.8;7.8]	0.287
**SRI-SS: summary scale**	60 [49.8;71.6]	62.3 [49.9;80.3]	+ 5.4 [-8.2;15.1]	0.144

*Abbreviations*: *SRI* Severe Respiratory Insufficiency questionnaire

*Note*: values are presented median and 25–75 percentiles. Wilcoxon test with *highlighting statistically significant differences between disease groups (*p* <0.05)

Surviving patients scored higher on the final SRI than in the questionnaire at inclusion (median difference =  + 5.4), although this difference was not statistically significant. The only difference that achieved statistical significance was related to the attendant symptoms and sleep subscale, with patients scoring more than 7 points higher in the final questionnaire (*p* = 0.002).

The differences in SRI questionnaire at 5 years within each disease group are described in supplementary table S2. There is some heterogeneity regarding the 5-year difference between diseases, with COPD and OHS showing a slight decrease in SRI score and the remainder an increase, although groups were too small to be compared.

### HRQoL at inclusion of survivors and deceased

Differences in SRI results at inclusion between survivors and deceased are depicted in Table 3. Survivors had significantly better median scores in the SRI summary score than deceased patients (59.6 vs 48.7; *p* = 0.004). These better scores were also seen in the following subscales: respiratory complaints, physical functioning, anxiety and social functioning, all with statistically significant changes, as can be seen in Table 3.

**Table 3:** Severe Respiratory Insufficiency questionnaire at inclusion for survivors and deceased (*n* = 104)

	**All (*n* = 104)**	**Survivors (*n* = 57; 54.8%)**	**Deceased (*n* = 47; 45.2%)**	***p*-value**
**SRI-RC: respiratory complaints**	59.4 [43.8;74.2]	65.6 [46.9;81.3]	56.3 [40.6; 67.9]	0.042*
**SRI-PF: physical functioning**	45.8 [26;66.7]	54.2 [35.4;70.8]	37.5 [16.7; 62.5]	0.003*
**SRI-AS: attendant symptoms and sleep**	50 [39.3;64.3]	50 [39.3;64.3]	50 [35.7; 62.5]	0.412
**SRI-SR: social relationships**	75 [62.5;91.7]	75 [62.5;91.7]	70.8 [62.5; 87.5]	0.389
**SRI-AX: anxiety**	40 [25;65]	45 [32.5;67.5]	35 [20;55]	0.044*
**SRI-WB: psychological well-being**	55.6 [41.7;71.5]	58.3 [45.7;72.2]	52.8 [33.3; 66.7]	0.145
**SRI-SF: social functioning**	56.3 [40.6;75]	65.6 [46.9;84.4]	50 [31.3;68.8]	< 0.001*
**SRI-SS: summary scale**	53.3 [44.9;68.2]	59.6 [49.2;71.7]	48.7 [38.4;63.2]	0.004*

*Abbreviations*: *SRI* Severe Respiratory Insufficiency questionnaire

*Note*: values are presented median and 25–75 percentiles. Mann–Whitney test with * highlighting statistically significant differences between disease groups (*p* < 0.05)

**Fig. 1:**
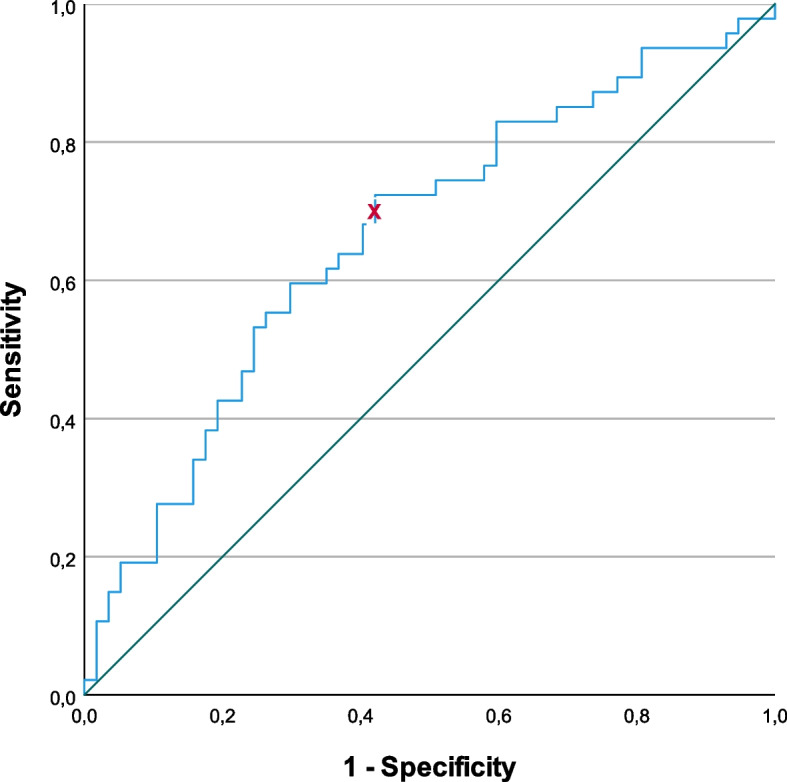
ROC curve (blue) of the SRI at inclusion’s ability in discriminating survivors (*n* = 57) from deceased (*n* = 47). The best cut-off was 56.2 (marked with red x) obtained by Youden method

The ROC curve analysis of the SRI summary score at inclusion and mortality is shown in Fig. 1. We found an area under the ROC curve (AUROC) of 0.663 [95% CI 0.557; 0.768] with a cut-off by Youden method of 56.2, corresponding to a specificity of 0.421 and a sensitivity of 0.702.

### Survival analysis

**Fig. 2:**
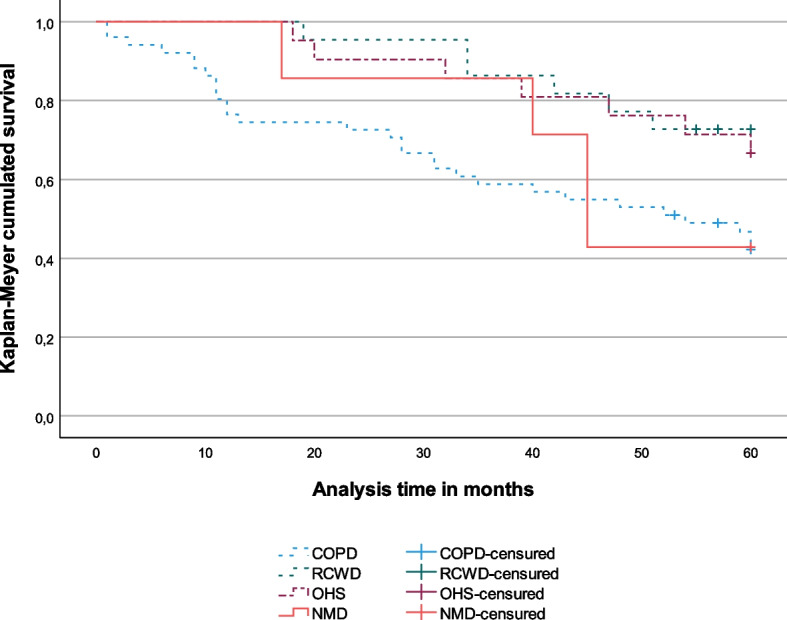
Kaplan Meyer survival analyses by disease group. Abbreviations: COPD, chronic obstructive pulmonary disease; OHS, obesity-hypoventilation syndrome; RCWD, restrictive chest wall disorders; NMD, neuromuscular disorders

We found a statistically significant difference (χ^2^(3) = 8.50, *p* = 0.037) of survival times between diseases. As shown in Fig. 2, at any point in time, survival in RCWD (mean [95% confidence interval] = 54 months [49.2; 58.7]) and OHS patients (52.9 months [46.7; 59]) was higher than for COPD patients (40.8 months [34.7; 46.9]).

## Discussion

Our study found that HRQoL measured by a condition-specific questionnaire at two points in a 5-year period in patients with long-term HMV remained stable in surviving patients. It also suggests that the SRI may be used as a predictor for 5-year mortality.

The study population reflects the most common diagnosis for CRF and has a distribution by disease similar to other studies from Portugal [2, 21, 23, 24] and Europe [3, 25]. As a real-life study, patients are less selected than RCTs and that is evident on the clinical characteristics of COPD patients, as obesity and concurrent OSA were not exclusion criteria. The considerable prevalence of these two characteristics has been described in other large real-life studies [3, 25–27].

Regarding HRQoL, it was found a median 5.4-point improvement in the SRI summary score and, although it was not statistically significant, it was above the estimated minimal clinical important difference for the SRI in patients with COPD, which is approximately 5 points [28]. The only statistically significant change was in the attendant symptoms and sleep subscale (median + 7.1 points). This may suggest that with longer treatment periods, patients are progressively more tolerant and report better quality of sleep.

It is also of note that, at inclusion, patients were already on HMV for a long period of time (median 43.5 months) and with very good adherence (8 h) and good control of hypoventilation (median pCO2 46), and this may also help explain the stability of results. In 2008, a study by Windisch and colleagues, [29] showed an improvement of HRQoL in patients with CRF following 1 month of HMV, which remained stable at this elevated level during the following year. A study by Duiverman et al. [30] found that COPD patients with rehabilitation and HMV had a non-significant decrease of 3.4 in HRQoL measured by the SRI at 24 months. In 2018, Markussen et al. [18] described an improvement in SRI summary scale (4.74), and social relationships (8.47), anxiety (7.94) and well-being (7.66) subscales. The improvements in the total score of SRI were seen in all disease groups, except in patients with COPD, who also had a reduction in five of seven SRI subscales [18]. Similarly to our study, HRQoL was measured in two time-points. It has been described that some patients with Amyotrophic Lateral Sclerosis and other severe chronic diseases develop what has been named a Response Shift, involving recalibration, reprioritization, and reconceptualization [31]. Through these mechanisms, patients with serious illnesses experience changes in those factors that they perceive as being important to maintenance of quality of life, thus recalibrate their expectations to more closely match reality [32].

This study also found that HRQoL is significantly impaired in home mechanically ventilated patients. The median score of the SRI-SS at inclusion (53.3) was approximately in the middle of the questionnaire’s scaling range. These results are in line with other studies in stable ventilated patients and reflect the severity of patients’ disease and limitation [33, 34].

Five-year mortality was considerable, being highest among COPD and NMD patients, mostly progressive diseases, and lowest in the RCWD group, reflecting the non-progressive nature of this group. In line with previous studies, the time-to-death on HMV varies widely across disease groups with CRF and is lower in progressive diseases [13, 35].

Within the study period, we found that survivors at 5 years had significantly better SRI-SS at inclusion than deceased patients. A Norwegian cohort study of HMV patients using a 6-year follow-up period concluded that SRI score was inversely associated with mortality, even after adjusting for other factors such as age, education, hours a day on HMV, time since initiation of HMV, disease category and comorbidities. Interestingly, our values are very similar to the summary scores reported by this study, where survivors had a mean SRI-SS of 60.0 and deceased of 48.4 [13]. Our study estimated a cut-off value of 56.2 for the SRI-SS as a threshold for a 5-year mortality.

These results need to be confirmed by further including larger samples to ascertain whether a single measurement might offer important information for the patient and physician in daily clinical practice and defining a threshold value related to mortality.

In a study of 56 stable HMV patients with COPD or tuberculous sequelae, the authors found that SRI was significantly predictive of mortality, independently of the physiological measures of low BMI, hypercapnia, and low pulmonary function.

Predicting future outcomes is an important feature in assessing HRQoL. Prognostic information from the SRI questionnaire might provide valuable knowledge on identification of an approaching terminal disease course and facilitate the developing of coping mechanisms, improving treatment plans and communication between involved health professionals, family members and patients.

There might be some potential limitations to this study. Firstly, the sample size was relatively small, specially to ascertain differences at 5 years between diseases. Nevertheless, our sample size was similar to other studies with the same scope and included more than half of the patients ventilated in the clinic in that period. Secondly, patients were established on HMV for a long median period of time at inclusion and HRQoL relates to that time and not at start of HMV. However, this methodology was adopted as authors aimed at assessing the evolution of HRQoL on patients already established on HMV, to ascertain if benefits are maintained in the long-term.

Thirdly, the authors did not evaluate other explaning factors for change in HRQoL such as level of autonomy, exacerbations, hospital admissions or comorbidities [36, 37]. Fourthly, measurements of HRQoL were made just in two points in time with a five-year difference. It would be interesting to have serial measurements (every 6 months, or each year for example) to study more thoroughly the evolution of HRQoL in HMV patients and to ascertain if there is an inflection point, where disease course leads to HRQoL decline and mortality might be predicted. Further research is needed with repeated measurements to address these issues. Nevertheless, the authors believe that this study is reliable in showing that HRQoL remains stable in surviving patients with HMV in the long-term and it may predict mortality in these patients. Strategies to optimize HRQoL need to be implemented in routine clinical practice as it may have a relevant impact not only on patients and families’ well-being, but also on patients’ survival.

## Conclusions

The results of this study indicate that HRQoL, measured by the SRI Questionnaire, remains stable in surviving patients with HMV at five years. These results hint that there may be reference values and thresholds in the SRI questionnaire that have implications in future outcomes such as mortality. They also suggest that SRI can be of important prognostic value and help predict the terminal phase of the disease course in patients with long-term HMV.

These results reinforce the importance of using HRQoL measurements in routine clinical practice.

## Supplementary Information

**Additional file 1: Table S1.** Baseline patients demographic, clinical and ventilation characteristics of survivors and deceased patients. **Table S2.** Differences in SRI questionnaire at 5 years within each disease group.

## Data Availability

The datasets used and analyzed during the current study are available from the corresponding author on reasonable request.

## Abbreviations

BMI: Body mass index
COPD: Chronic obstructive pulmonary disease
CRF: Chronic respiratory failure
EPAP: Expiratory positive airway pressure
FVC: Forced vital capacity
FEV1: Forced expiratory volume in one second
HMV: Home mechanical ventilation
HRQoL: Health-related quality of life
IPAP: Inspiratory positive airway pressure
NIV: Noninvasive ventilation
NMD: Neuromuscular disorders
OHS: Obesity-hypoventilation syndrome
RCWD: Restrictive chest wall disorders
SRI: Severe respiratory insuffiency questionnaire
