# Supplementary material for: Long-term health-related quality of life in patients on home mechanical ventilation
Source: BMC Pulm Med. 2022 Nov 22;22:433. doi: 10.1186/s12890-022-02236-z (PMC9682680; doi:10.1186/s12890-022-02236-z)
Supplement: Supplementary file 1 — Additional file 1: Table S1. Baseline patients demographic, clinical and ventilation characteristics of survivors and deceased patients. Table S2. Differences in SRI questionnaire at 5 years within each disease group. [file 12890_2022_2236_MOESM1_ESM.docx]

Table S1 - Baseline patients demographic, clinical and ventilation characteristics of survivors and deceased patients

|  | **All (N=104)** | **Survivors (n=57; 54.8%)** | **Deceased (n=47; 45.2%)** | **p-value** |
| --- | --- | --- | --- | --- |
| **Sex, n (%)** |  |  |  |  |
| **Male** | 59 (56.7) | 29 (50.9) | 30 (63.8) | 0.185 ^c^ |
| **Age (years)** | 69 [61; 77] | 68 [61;73.5] | 74 [59;81] | 0.056 ^d^ |
| **BMI ^a^** | 30.5 [25.3;37.3] | 31.8 [26.7;38.1] | 29 [24.9;35.5] | 0.116 ^d^ |
| **Disease, n (%)** |  |  |  | 0.097^e^ |
| **COPD** | 51 (49.0) | 22 (38.6) | 29 (61.7) |  |
| **RCWD** | 22 (21.2) | 16 (28.1) | 6 (12.8) |  |
| **OHS** | 21 (20.2) | 14 (24.6) | 7 (14.9) |  |
| **NMD** | 7 (6.7) | 3 (5.3) | 4 (8.5) |  |
| **Other** | 3 (2.9) | 2 (3.5) | 1 (2.1) |  |
| **FEV1 (% predicted)^b^** | 42.5 [27.8;57.7] | 46.5 [32.3;58] | 38 [26.1;54.3] | 0.175 ^d^ |
| **FVC%(% predicted) ^b^** | 59.1 [46.9;72.5] | 60.9 [46.2;75.8] | 57.1 [46.9;67.3] | 0.381 ^d^ |
| **pH** | 7.4 [7.4;7.4] | 7.4 [7.4;7.4] | 7.4 [7.4;7.4] | 0.984 ^d^ |
| **pO2 mmHg** | 69.4 [64.5;77.4] | 69.7 [65.2;77.6] | 68.2 [63;77] | 0.293 ^d^ |
| **pCO2 mmHg** | 46 [42.9;49.1] | 45 [42;48], | 47.6 [44;51], 36.2-63.1 | 0.055 ^d^ |
| **HCO3 mmol/L** | 28.3 [26.5;30.3] | 28.2 [26.1;30.3] | 28.4 [27;30.3] | 0.416 ^d^ |
| **Months on HMV** | 43.5 [22;85.5] | 45 [22;91.5] | 43 [19;76] | 0.364 ^d^ |
| **HMV Usage (hours/day)** | 8 [6; 9] | 8 [6;8.3] | 8 [7;9] | **0.020** ^d^ |

Abbreviations: COPD, chronic obstructive pulmonary disease; OHS, obesity-hypoventilation syndrome; RCWD, restrictive chest wall disorders; COPD+OSA, combined COPD and obstructive sleep apnea; NMD, neuromuscular disorders; BMI, body mass index; HMV, home mechanical ventilation; FVC, forced vital capacity; FEV1, forced expiratory volume in one second; IPAP, inspiratory positive airway pressure; EPAP, expiratory positive airway pressure

Note: values are presented as n (%) or median and 25-75 percentiles

^a^ 2 missing ^b^ 6 missing ^c^ Chi-square test ^d^ Man-Whitney test ^e^ Fisher-Freeman-Halton exact test

Table S2 - Differences in SRI questionnaire at 5 years within each disease group

| **Scale** | **COPD (n=20)** | **RCWD (n=16)** | **OHS (n=13)** | **NMD (n=3)** | **others (n=2)** |
| --- | --- | --- | --- | --- | --- |
| **SRI-RC: respiratory complaints** | 3.1 [-6.3;23.4] | 4.7 [-6.3;15.6] | 0 [-15.6;12.9] | 9.4 [0;40.6] | 3.1 [3.1;3.1] |
| **SRI-PF: physical functioning** | -10.4 [-20.8;12.5] | -0.4 [-14.6;18.8] | -8.3 [-20.8;14.2] | 0 [-8.3;8.3] | 12.5 [-8.3;33.3] |
| **SRI-AS: attendant symptoms and sleep** | 0 [-7.1;25] | 7.1 [-3.6;21.4], | 7.1 [0;21.4] | 21.4 [-3.6;25] | 48.2 [42.9;53.6] |
| **SRI-SR: social relationships** | 10.4 [-8.3;33.3] | 4.2 [-10.4;20.8] | -1.7 [-16.7;12.5] | 8.3 [-37.5;8.3] | 12.5 [12.5;12.5] |
| **SRI-AX: anxiety** | -5 [-17.5;2.5] | 7.5 [-10;30], | 8.7 [0;10] | 5 [-10;20] | 12.5 [0;25] |
| **SRI-WB: psychological well-being** | 5.6 [-8.3;19.4] | 8 [-12.5;23.6] | 2.8 [-16.7;19.4] | -5.6 [-11.1;11.1] | 18.1 [8.3;27.8] |
| **SRI-SF: social functioning** | -0.6 [-18.8;13.4] | 1.6 [-11.6;18.1], | -8.9 [-18.3;-6.3] | 0 [-25;1.8] | 10.9 [3.1;18.8] |
| **SRI-SS: summary scale** | -0.4 [-5.8;14.3], | 7.9 [-3.1;15.2], | -1.6 [-13;15] | 6.1 [-6.6;8.8] | 16.8 [8.8;24.9] |

Abbreviations: COPD, chronic obstructive pulmonary disease; OHS, obesity-hypoventilation syndrome; RCWD, restrictive chest wall disorders; COPD+OSA, combined COPD and obstructive sleep apnea; NMD, neuromuscular disorders

Note: values are presented as median and 25-75 percentiles
